# Development of Antifouling Polysulfone Membranes by Synergistic Modification with Two Different Additives in Casting Solution and Coagulation Bath: Synperonic F108 and Polyacrylic Acid

**DOI:** 10.3390/ma15010359

**Published:** 2022-01-04

**Authors:** Katsiaryna S. Burts, Tatiana V. Plisko, Mikael Sjölin, Goncalo Rodrigues, Alexandr V. Bildyukevich, Frank Lipnizki, Mathias Ulbricht

**Affiliations:** 1 Institute of Physical Organic Chemistry, National Academy of Sciences of Belarus, 220072 Minsk, Belarus; katyaburt@gmail.com (K.S.B.); uf@ifoch.bas-net.by (A.V.B.); 2 Department of Chemical Engineering, Lund University, 221 00 Lund, Sweden; mikael.sjolin@chemeng.lth.se (M.S.); goncaloncrodrigues@ist.utl.pt (G.R.); frank.lipnizki@chemeng.lth.se (F.L.); 3 Department of Bioengineering, Instituto Superior Técnico, 1049-001 Lisbon, Portugal; 4 Lehrstuhl für Technische Chemie II, Universität Duisburg-Essen, 45141 Essen, Germany; mathias.ulbricht@uni-essen.de

**Keywords:** membrane modification, ultrafiltration, polysulfone, Synperonic F108, polyacrylic acid, hemicellulose, wastewater treatment, fouling

## Abstract

This study deals with the development of antifouling ultrafiltration membranes based on polysulfone (PSF) for wastewater treatment and the concentration and purification of hemicellulose and lignin in the pulp and paper industry. The efficient simple and reproducible technique of PSF membrane modification to increase antifouling performance by simultaneous addition of triblock copolymer polyethylene glycol-polypropylene glycol-polyethylene glycol (Synperonic F108, M_n_ =14 × 10^3^ g mol^−1^) to the casting solution and addition of polyacrylic acid (PAA, M_n_ = 250 × 10^3^ g mol^−1^) to the coagulation bath is proposed for the first time. The effect of the PAA concentration in the aqueous solution on the PSF/Synperonic F108 membrane structure, surface characteristics, performance, and antifouling stability was investigated. PAA concentrations were varied from 0.35 to 2.0 wt.%. Membrane composition, structure, and topology were investigated by Fourier-transform infrared spectroscopy (FTIR), X-ray photoelectron spectroscopy (XPS), atomic force microscopy (AFM), and scanning electron microscopy (SEM). The addition of PAA into the coagulation bath was revealed to cause the formation of a thicker and denser selective layer with decreasing its pore size and porosity; according to the structural characterization, an interpolymer complex of the two additives was formed on the surface of the PSF membrane. Hydrophilicity of the membrane selective layer surface was shown to increase significantly. The selective layer surface charge was found to become more negative in comparison to the reference membrane. It was shown that PSF/Synperonic F108/PAA membranes are characterized by better antifouling performance in ultrafiltration of humic acid solution and thermomechanical pulp mill (ThMP) process water. Membrane modification with PAA results in higher ThMP process water flux, fouling recovery ratio, and hemicellulose and total lignin rejection compared to the reference PSF/Synperonic F108 membrane. This suggests the possibility of applying the developed membranes for hemicellulose concentration and purification.

## 1. Introduction

The pulp and paper industry is one of the most polluting and water consuming industries in the world. Wastewater in the pulp and paper industry is usually highly loaded with organic and inorganic compounds, such as lignin, cellulose, hemicellulose, phenols, fatty acid, resins, and others [1]. To reduce the consumption of water, membrane technologies (in particular, ultra- and microfiltration) are used for the dewatering of the waste solution formed after the completion of pulping. Valuable substances contained in wood (lignin, hemicellulose, water-soluble aromatic compounds, polymers) are released during delignification and dissolve in technological media (thermomechanical pulp mill (ThMP) process water) during the production of thermomechanical cellulose [2,3]. An urgent problem is the insufficient use of cellulose-processing products (lignin and hemicellulose) [2,3,4]. Most of these substances end up in wastewater from the pulp and paper industry, which entails costly wastewater treatment. Reducing the amount of residual substances in wastewater reduces the cost of wastewater treatment and the load on treatment plants [3,4]. At the same time, the isolated substances—for instance, hemicellulose—can be used as renewable materials for the production of special reagents and biofuels. For instance, galactoglucomannan is the most common polysaccharide contained in hemicelluloses, and ThMP process water can be used for the production of food packaging films, hydrogels, emulsion stabilizers in the production of alcoholic beverages, and as an additive for increasing paper strength [4].

Ultrafiltration is an effective method of concentration and purification of lignin and hemicellulose and water purification in the pulp and paper industry. The advantage of ultrafiltration is the high selectivity of the separation process, low energy consumption, ecological safety, and lack of necessity of using additional reagents [2,3,4,5,6].

However, fouling is considered as one of the main drawbacks of using membrane technologies. Membrane fouling occurs due to the adhesion or adsorption of solid particles, colloidal and oligo-/polymer compounds on the membrane surface [7]. Fouling causes the impairment of membrane performance, which is manifested in membrane flux, thus decreasing lifetime and increasing cost [8]. The adsorbed layer of dissolved substances on the membrane surface often has to be removed using aggressive chemicals to recover membrane performance [9]. However, chemical cleaning may result in membrane damage because of the destruction of the selective layer or changes in the structure and composition of the membrane matrix. Thus, there is an urgent need for the development of antifouling membranes for application in the pulp and paper industry for wastewater treatment and the concentration and purification of lignin and hemicellulose.

In order to decrease fouling, polymer membranes are modified by increasing their hydrophilicity, decreasing surface roughness, and charging the membrane selective layer [7,10]. Membrane modification by neutral hydrophilic polymers is an effective way to mitigate membrane fouling. For instance, polyvinyl pyrrolidone (PVP) and polyethylene glycol (PEG) of different molecular weights are used as additives in the casting solution upon membrane preparation [11,12,13,14,15,16]. It was demonstrated that amphiphilic block copolymers, when added to the casting solution upon membrane preparation, can tune the membrane pore structure and increase membrane antifouling performance due to an increase in membrane hydrophilicity. For instance, the block copolymer of polyethylene glycol and polypropylene glycol Pluronic F-127 was found to be effective in membrane hydrophilization as well as in the reduction of membrane fouling and enhancement of the flux recovery ratio after bovine serum albumin ultrafiltration [17,18].

Another cheap and easy to perform one-step method to increase membrane surface hydrophilicity and to introduce charged groups to the membrane selective layer is to use a polyelectrolyte as an additive in the coagulation bath upon membrane preparation by non-solvent-induced phase separation (NIPS) [19,20,21,22]. It was shown that an introduction of polyelectrolytes (copolymers of acrylamide and 2-acryloxyethyltrimethylammonium chloride [19,21], copolymers of acrylamide and sodium acrylate [20,22], polyacrylic acid (PAA) [23], and polyethyleneimine (PEI) [24]) into the coagulation bath leads to the immobilization of macromolecules in the selective layer because of the interlacing of chains of membrane-forming polymers and polyelectrolytes during membrane formation by NIPS. This occurs due to the diffusion of polyelectrolyte macromolecules inside the nascent polymer membrane. It was found that the immobilization of polyelectrolytes may significantly improve membrane antifouling performance due to the presence of hydrophilic groups, which are also capable of ionization, in the structure of the membrane selective layer [19,20,21,22,23,24]. The polyelectrolyte addition to the coagulation bath was revealed to influence the kinetics of phase separation, due to the rise of coagulant viscosity, which results in a delayed demixing mechanism of the phase inversion, which is confirmed by a significant increase in membrane formation time [19,20,21,22].

PAA is a commonly used polyelectrolyte for membrane modification, which can tailor a negative charge at a high pH. For instance, He et al. [23] studied the modification of the poly(vinylidene fluoride)/PVP ultrafiltration membrane by a reaction-enhanced surface segregation method using polyacrylic acid (PAA). PVP was applied as an additive to the casting solution, and PAA was used as an additive to the coagulation bath upon membrane preparation via NIPS. It was shown that this modification method allows decreasing the water contact angle down to 24 °C and increasing the membrane flux recovery ratio from 34% and 45% for the reference membrane to 90–96% and 83–95% for PAA-modified membranes for the ultrafiltration of an oil–water emulsion and BSA solution, respectively.

It was reported that the surface modification of polysulfone (PSF) ultrafiltration membranes via the addition of copolymer of acrylamide and sodium acrylate to the coagulation bath upon membrane preparation via NIPS resulted in the improvement of hydrophilicity and change of zeta potential of the membrane selective layer, featuring higher negative values at pH 7-9 [20]. It was revealed that the modified membrane showed better separation and antifouling performance in the ultrafiltration of the humic acid solution and surface water compared to the reference membrane [20].

The aim of the present work was a detailed and systematic study of the method of modification of PSF ultrafiltration membranes by the simultaneous addition of triblock copolymer polyethylene glycol (PEG)-polypropylene glycol (PPG)-polyethylene glycol (PEG) (Synperonic F108) to the casting solution and PAA to the coagulation bath during membrane preparation by NIPS. The novelty of this study is that for the first time, the coupled effect of the modification of PSF ultrafiltration membranes by the addition of Synperonic F108 to the casting solution and the addition of PAA to the coagulation bath was studied. The value of the proposed modification technique is the possibility of significantly improving membrane rejection and increasing hydrophilicity, charge, and antifouling performance by the simple addition of commercially available modifiers (Synperonic F108 and PAA) to the casting solution and coagulation bath, which does not require a significant change of the technological process of membrane preparation. A thorough investigation of the effect of PAA concentration in the coagulation bath on the PSF/Synperonic F108 membrane pore structure, hydrophilic–hydrophobic balance, zeta potential, topology, performance, and antifouling stability was performed. The composition of the selective layer was explored by Fourier-transform infrared (FTIR) spectroscopy and X-ray photoelectron spectroscopy (XPS). The membrane structure was investigated by scanning electron microscopy (SEM) and atomic force microscopy (AFM). Physico-chemical properties of the selective layer were studied by water contact angle and zeta potential measurements. The antifouling stability of PSF/Synperonic F108 and PSF/Synperonic F108/PAA membranes was evaluated in the ultrafiltration of model humic acids solution and thermomechanical pulp mill (ThMP) process water.

## 2. Materials and Methods

### 2.1. Materials

Polysulfone (PSF, Ultrason S 6010, Mn = 55 × 10^3^ g mol^−1^, BASF) was used as a membrane-forming material. Triblock copolymer polyethylene glycol (PEG)-polypropylene glycol (PPG)-polyethylene glycol (PEG) (PEG-PPG-PEG, Synperonic© F108, M_n_ = 14 × 10^3^ g mol^−1^, 80% ethylene glycol (EG) blocks, 20% propylene glycol (PG) blocks, Serva, Germany) was used as an additive to the casting solution for membrane preparation. N,N-dimethylacetamide (DMAc, 99.8% degree of purity, BASF, Germany) was utilized as a solvent. All abovementioned reagents were used directly without prior purification.

Polyacrylic acid (PAA, M_n_ = 250 × 10^3^ g mol^−1^, Sigma-Aldrich, St. Louis, MO, USA) aqueous solutions were used as coagulation bath upon membrane preparation by non-solvent-induced phase separation (NIPS). PAA concentrations in the coagulation bath were 0.35; 0.5; 0.7; 1.0; 1.2; 1.5; and 2.0 wt.%. To obtain polyelectrolyte aqueous solutions, a certain amount of PAA was dissolved in distilled water for 2 h using a magnetic stirrer at 20 °C.

Membrane separation performance (rejection coefficients and flux) was examined using polyvinylpyrrolidone (PVP K-30, M_n_ = 40 × 10^3^ g mol^−1^, Sigma-Aldrich) and lysozyme (Lys, M_n_ = 14.3 × 10^3^ g mol^−1^, Sigma-Aldrich) aqueous solutions. Anhydrous sodium monobasic phosphate (KH_2_PO_4_) and sodium dibasic phosphate trihydrate (K_2_HPO_4_∙3H_2_O) were supplied by Sigma-Aldrich and used for preparation of phosphate buffer (pH 7.2).

To obtain 0.005 wt.% humic acids (HAs) model solution, fertilizer “Hydrohumin” (Biochem, Svisloch, Belarus, 50 wt.% HAs) was diluted by tap water. This HAs model solution was used to assess the antifouling and separation performance of the initial and developed membranes.

The thermomechanical pulp mill (ThMP) process water (Stora Enso, Kvarnsveden Mill, Borlänge, Sweden) was used for the study of separation and antifouling performance of developed membranes. FennoCide BZ26 (Kemira, Helsinki, Finland) was applied as a biocide. The concentration of total solids was 6.41 g L^−1^ and had an ash content of 1.22 mg g^−1^. The concentration of hemicelluloses and total lignin were 2.01 g L^−1^ and 0.89 g L^−1^, respectively.

### 2.2. Flat-Sheet Ultrafiltration Membrane Preparation

Preparation of casting polymer solutions was described previously in [14]. A casting solution of 20 wt.% PSF and 7 wt.% Synperonic F108 in DMAc was applied on a glass plate using a blade with a thickness of 250 µm. The composition of the casting solution was selected according to previous studies [17,18]. Polymer film on the glass plate was then immersed into the coagulation bath containing distilled water or 0.35–2.0 wt.% PAA aqueous solution. Polyelectrolyte concentration in the coagulation bath and appropriate membrane designations are described in Table 1. For comparison of physico-chemical properties of membrane selective layer, an additional reference membrane from the casting solution containing 20 wt.% PSF and 80 wt. % DMAc was prepared using distilled water as a coagulation bath (PSF-0). Membranes were kept in distilled water for 48 h prior to ultrafiltration tests.

### 2.3. Determination of Viscosity of PAA Aqueous Solutions

Kinematic viscosity (ν, mm^2^ s^−1^) of polyelectrolyte solutions was investigated with suspended-level viscometer (d = 0.56 mm) at room temperature (20 °C). The relative error of measurements was 1%.

### 2.4. Study of Membrane Formation Time

Membrane formation time via NIPS was measured as the period of time between the immersion of a polymer film into the coagulation bath and the moment when formed membrane spontaneously peels from the glass substrate. Five membrane samples of each type were prepared, and membrane formation time was determined and averaged.

### 2.5. Study of Membrane Composition

#### 2.5.1. FTIR Spectroscopy

Composition of membrane selective and bottom layers was studied by Fourier-transform infrared spectroscopy (FTIR) using Nicolet Is50 IR Fourier spectrometer (Thermo Scientific™, Waltham, MA, USA) in the frequency range of 400–4000 cm^−1^ with an accuracy of 0.01 cm^−1^.

#### 2.5.2. XPS Study

X-ray photoelectron spectroscopy (XPS) measurements were performed with the instrument PHI 5000 Versa Probe ІІ (Physical Electronics, Inc. /PHI/, Chanhassen, MN USA), using monochromatized Al-Kα X-ray source (1486.6 eV).

### 2.6. Water Contact Angle

The water contact angle (CA) of the membrane selective layer surface was determined by the attached bubble method, described in [25]. Measurements were carried out for at least three membrane samples of each type. Five different places on the membrane surface of each sample were investigated, and the average water contact angle was calculated. Measurement errors did not exceed ±2°.

### 2.7. Membrane Morphology

Scanning electron microscopy (SEM) measurements of the surfaces of membrane selective layer were performed with the instrument Apreo S LoVac (Thermo Scientific™, Waltham, MA, USA). Samples were prepared in two different manners, either via freeze-drying of the water-wet membrane using the FreezeDryer Alpha 1–4 (Christ, Osterode am Harz, Germany) or via stepwise solvent exchange from water via water/ethanol mixtures to ethanol and finally to hexane and ultimately air drying. The sputtering with an alloy containing 80% Au and 20% Pd was done using a K-550 sputter coater (Emitech, Quorum Technologies Ltd, Laughton, UK).

### 2.8. Membrane Topography

To study the membrane surface structure, an atomic force microscope NT-MDT NTegra Maximus (NT-MDT Spectrum Instruments, Zelenograd, Russia) equipped with standard silicon cantilevers and a rigidity of 15 N m^−1^ was used. Membrane samples were impregnated with 50 wt.% aqueous solution of glycerol and dried at ambient temperature for at least three days prior to AFM studies.

### 2.9. Zeta Potential

To measure zeta potential of the membrane selective layer, SurPASS 2 instrument (Anton Paar, Graz, Austria) was used. Methodology of measurements was defined in [20].

### 2.10. Membrane Ultrafiltration Performance

Membrane pure water flux (PWF, L m^−2^ h^−1^) was determined as a permeate volume passed through the unit of membrane area in the unit of time [11,19,20,21,22]. The ultrafiltration studies were described in detail in [17]. After measurements of membrane PWF, 0.3 wt.% aqueous solution of PVP K-30 or 0.5 wt.% lysozyme solution in a phosphate buffer (pH 7.2) was used. Flux (J, L m^−2^ h^−1^) and rejection (R, %) of the reference PFS/Synperonic F108 and PSF/Synperonic F108/PAA-modified membranes were measured after 40 min of ultrafiltration at 1 bar and ambient temperature. Rejection of lysozyme and PVP K-30 was investigated by measuring their concentrations using UV–vis spectrophotometer (Metertech UV–VIS SP 8001, Taipei, Taiwan) at a wavelength of 280 nm and Rayleigh interferometer, correspondingly. The rejection was calculated using Equation (1):

(1) $$R=\left(1–\frac{C_{p}}{C_{f}}\right)\;\cdot \;100,$$

where *C_p_* and *C_f_* are concentrations of lysozyme or PVP K-30 in permeate and feed solution, respectively. For measurement of membrane ultrafiltration performance, at least five different membrane samples were tested (each three times), and the average flux and rejection were calculated.

### 2.11. Membrane Antifouling Performance

#### 2.11.1. Filtration of HAs Aqueous Solution

Antifouling performance of reference SA-0 and PAA-modified membranes was studied during filtration of 0.005 wt.% HAs solution in tap water. First, membrane was cleaned from impregnated glycerol followed by the pure water flux measurements at transmembrane pressure of 2 bar. After that, water was replaced by the HAs solution and filtered for 1 h. HAs flux (J_HAs_, L m^−2^ h^−1^) and rejection were measured every 10 min for 1 h. The next step was to clean membrane with MilliQ water after HAs filtration and to determine membrane pure water flux upon 30 min. Three membrane samples were tested to evaluate membrane antifouling performance toward HAs aqueous solution. The flux recovery ratio (FRR, %) and total flux decline ratio (DT, %) were evaluated according to Equations (2) and (3), correspondingly:

(2) $$\mathrm{FRR}=\left(\frac{\mathrm{JWF}}{\mathrm{PWF}}\right)\;\cdot \;100,$$

(3) $$\mathrm{DT}=\left(\frac{\mathrm{PWF}-J_{\mathrm{HAs}}}{\mathrm{PWF}}\right)\;\cdot \;100,$$

where JWF is water flux of the rinsed membrane, L m^−2^ h^−1^, and J_Has_ is flux of HAs solution, L m^−2^ h^−1^.

Feed solution characteristics are presented in Table 2.

The optical density of HAs feed solution and permeate were analyzed using UV–vis spectrophotometer at the wavelength of 400 nm. Iron content both in feed solution and in permeate was determined by an inductively coupled plasma atomic emission spectrometer (Vista PRO, Varian, Palo Alto, CA, USA).

#### 2.11.2. Assessment of Transport Properties and Antifouling Stability during Ultrafiltration of ThMP Process Water

The ultrafiltration experimental setup used for the filtration of a recirculating batch of ThMP process water in crossflow mode is described in detail in [21,22].

The experimental procedure prior to ThMP exposure started with a conditioning step to clean the membranes from preservatives. This chemical cleaning was conducted for 1 h at 50 °C using Ultrasil 10 (Ecolab AB, Älvsjö, Sweden) as an alkaline cleaning agent with a dosing concentration of 1% (*w*/*w*). This cleaning cycle was performed at a constant CFV of 0.5 ms^−1^ and at a TMP of 2 bar. The same cleaning protocol and conditions were also used after the ultrafiltration of ThMP process water. During the ultrafiltration of the wastewater from the pulp industry, temperature and CFV were maintained constant at 70 °C and 0.3 m s^−1^. The permeate fluxes were logged for the TMP of 1, 3, and 5 bar, then normalized with respect to pressure, and calculated as average permeance according to Equation (4):

(4) $$P=\frac{V}{A\;\cdot \;t\cdot \mathrm{TMP}},$$

where P is membrane permeance, L m^−2^ h^−1^ bar^−1^; V is volume of permeate, L; A is effective membrane area, m^2^; t is time, h; and TMP is transmembrane pressure, bar.

The setup was rinsed with several batches of deionized water between each process step and followed by PWF measurements. The PWF measurements were conducted with deionized water after the ultrafiltration of ThMP process water as well as after each cleaning and conditioning step. The PWF measurements were performed at 30 °C and constantly kept CFV of 0.3 m s^−1^, as the TMP was set stepwise at 1, 2, and 3 bar when the sampling also occurred. All permeate flux measurements were performed after achieving flux steady state. Three membrane samples were tested to evaluate membrane antifouling performance and transport properties during ultrafiltration of ThMP process water, and average values of membrane pure water flux, permeance, and rejection were calculated.

#### 2.11.3. Evaluation of Membrane Fouling and Cleaning Efficiency

The fouling severity on the membrane surfaces was determined using FRRs after deionized water rinsing (FRR_rinse_, %), and the efficiency of the applied cleaning protocol was determined in accordance with the FRRs after cleaning (FRR_clean_, %). These two parameters were defined by Equations (5) and (6):

(5) $${\mathrm{FRR}}_{\mathrm{rinse}}=\frac{P_{\mathrm{rinse}}}{P_{0}}\cdot 100\%,$$

(6) $${\mathrm{FRR}}_{\mathrm{clean}}=\frac{P_{\mathrm{clean}}}{P_{0}}\cdot 100\%,$$

where P_0_ is pure water permeance before the ultrafiltration of ThMP process water, L m^−2^ h^−1^ bar^−1^, and P_rinse_ and P_clean_ are pure water permeances before and after the second chemical cleaning step, correspondingly, L m^−2^ h^−1^ bar^−1^.

#### 2.11.4. Rejection Coefficients in ThMP Filtration Process

To determine the concentrations and rejections of hemicelluloses, mannan was used as an indicator sugar for hemicellulose, since galactoglucomannan is the most common hemicellulose found in softwood [26]. The rejection (R, %) of mannan and lignin compounds was determined using Equation (1).

#### 2.11.5. Analytical Methods

The following analyses were conducted according to the standardized NREL method [27,28,29].

Determination of conductivity of ThMP process water feed and permeate solutions as well as content of hemicellulose, lignin, total solids, and ash in samples before and after ThMP process water ultrafiltration was described previously [21,22].

## 3. Results and Discussion

### 3.1. Study of the Membrane Composition

#### 3.1.1. FTIR Studies

**Figure 1:**
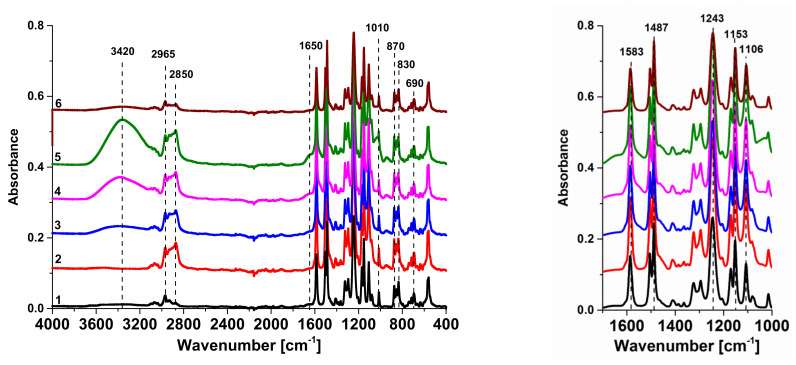
FTIR spectra of the selective layers (1–5) and a bottom layer (6) of PSF, PSF/Synperonic F108, and PSF/Synperonic F108/PAA membranes: 1—PSF-0; 2—SA-0; 3—SA-0.35; 4—SA-1.0; 5, 6—SA-1.5.

The composition of selective and bottom layers of the PSF-0, PSF/Synperonic F108 membrane, and PAA-modified membranes was investigated by FTIR (Figure 1). The main peaks in spectra both for reference and modified membranes are attributed to the PSF membrane matrix as the concentration of other components is much lower.

The peaks at 1153, 1583, 1487, 1106, 1243, 2850, and 2965 cm^−1^ correspond to PSF and were discussed previously in several studies [30,31,32,33,34]. Peaks at 690 cm^−1^, 830, and 870 cm^−1^ are related to out of plane aromatic bending vibrations of PSF [31]. The band at 1100 cm^–1^ is attributed to the stretching vibration of the sulfoxide group [32].

The increase in the intensity of the peak at 2850 cm^−1^ assigned to the stretching vibrations of C-H bonds in the FTIR spectra for the selective layer of the SA-0 membrane is due to the presence of additional methyl and methylene groups from Synperonic F108 (2 in Figure 1). It is worth noting that the intensity of this peak practically does not change when PAA is added to the coagulation bath, indicating the presence of Synperonic F108 in the selective layer of modified membranes (3–5 in Figure 1). The intensity of the peak at 2850 cm^−1^ is lower for the FTIR spectrum of the bottom layer of the SA-1.5 membrane compared to the selective layer, indicating that fewer Synperonic F108 molecules are immobilized (6 in Figure 1). However, no additional bands confirming the incorporation of Synperonic F108 are observed in the FTIR spectra. Similar results were obtained when PSF and PES membranes were modified by the addition of the PEG-PPG-PEG block copolymer Pluronic F127 (M_n_ = 1.26 × 10^4^ g mol^−1^, 70% EG units, 30% PG units) to the casting solution [17,34]. This is due to overlapping of C-O-C group stretching vibrations of Synperonic F108 and PSF at 1106 cm^−1^. The indirect confirmation of the presence of Synperonic F108 is the ratio between the area of characteristic peaks of Synperonic F108 (1106 cm^−1^) to those of PSF (1585 cm^−1^). This ratio was found to be 0.87 for the selective layer of the PSF-0 membrane, 1.14 for SA-0, 1.1 for SA-0.35, 1.1 for SA-1.0, and 1.26 for SA-1.5. According to these ratios, it can be concluded that Synperonic F108 is immobilized in the membrane selective layer when added to the casting solution. However, the ratio of the peak areas for the bottom layer of the SA-1.5 membrane is 1.08, which confirms immobilization of fewer Synperonic F108 molecules.

The absorption peak at 1650 cm^−1^ is attributed to the vibrations of the С=О in the carboxylic groups of PAA on the selective layer surface of modified membranes (3–5 in Figure 1) [35]. It was found that FTIR spectra of the selective layer of modified membranes with 0.35–1.5 wt.% PAA in the coagulation bath feature a wide absorption peak in the range of 3000–3600 cm^−1^ with the maximum at 3420 cm^−1^. This peak is attributed to the O–H groups involved in the formation of intermolecular hydrogen bounds of the carboxylic groups of PAA and the ether oxygen atom of monomer units of Synperonic F108 on the surface of the selective layer or intramolecular hydrogen bonds between OH- groups of PAA macromolecules (3–5 in Figure 1). It was reported that PAA readily forms an interpolymer complex driven by hydrogen bond formation with PEG-PPG-PEG block copolymers as well as with polyethylene oxides in aqueous solutions [36,37,38]. It is possible that such an interpolymer complex can be formed on the selective layer surface of PSF/Synperonic F108/PAA membranes.

The presence of the peaks at 3420 cm^−1^ and 1650 cm^−1^ confirms the immobilization of PAA macromolecules in the selective layer when polyelectrolyte aqueous solution is used as a coagulation bath via NIPS (Figure 1). An increase in PAA concentration was found to yield an increase in the intensity of these peaks in the selective layer surface spectra (3–5 in Figure 1). It must be the result of immobilization of the larger number of polyelectrolyte macromolecules on the selective layer surface when PAA concentration increases in the coagulation bath. However, no vibration bands attributed to the PAA macromolecules on the bottom layer surface were found (6 in Figure 1). Similar results were obtained in our previous studies [19,20]. The absence of polyelectrolyte chains on the membrane bottom layer surface is associated with the fact that the bottom layer is blocked by the glass plate, and in-diffusion of PAA macromolecules in the nascent cast polymer film occurs exclusively from the side of the selective layer upon membrane preparation by NIPS. Moreover, contact of the polymer casting solution results in the formation of a partly precipitated gel-like selective layer, and the diffusion of solvent, non-solvent, and PAA macromolecules inside the polymer film happens through this barrier layer. PAA features lower the diffusion coefficient compared to the solvent and non-solvent due to a much higher molecular weight [19,20]. It is expected that the greatest part of the PAA macromolecules is immobilized on the selective layer surface as well as in the vicinity of the selective layer. A significant decline in the concentration of PAA macromolecules is expected to be formed across the thickness of the membrane from the top to the bottom.

#### 3.1.2. XPS Studies

**Figure 2:**
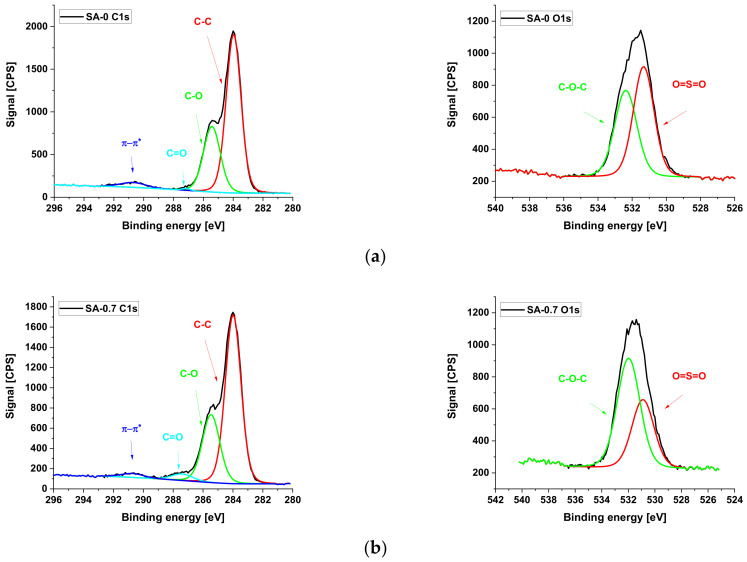

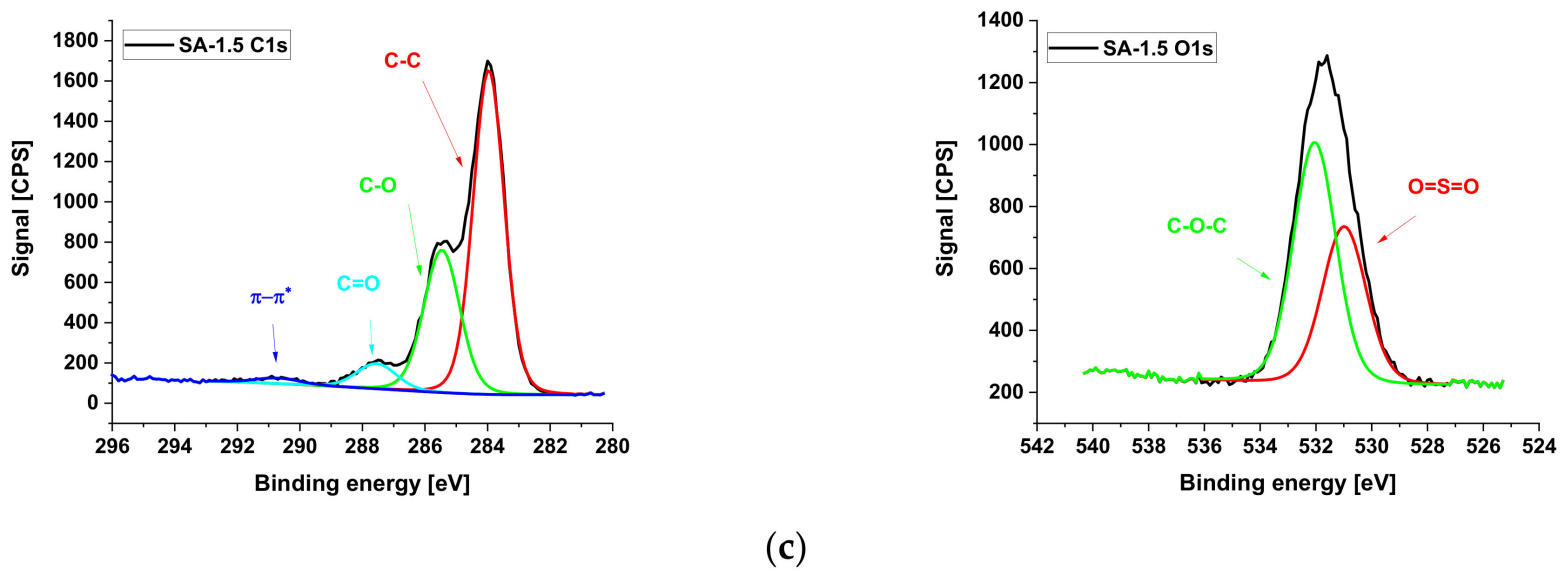
XPS spectra analysis of the selective layer of PSF/Synperonic F108 and PSF/Synperonic F108/PAA membranes: (**a**)—SA-0; (**b**)—SA-0.7; (**c**)—SA-1.5.

The composition of the selective layer surface was further studied using XPS (Table 3, Figure 2). It was found that the introduction of Synperonic F108 to the casting solution yields an increase in the atomic percent concentration of oxygen for the SA-0 membrane compared to the PSF-0 membrane. The increased level of oxygen results from the ether and hydroxyl groups of Synperonic F108 entrapped in the membrane selective layer (Table 3) [39,40]. It was revealed that an addition of 0.7 wt.% of PAA to the coagulation bath increased the atomic percent of oxygen of the surface of the selective layer from 14 to 18.5%. This is attributed to the appearance of carboxylic groups on the membrane surface due to the immobilization of PAA macromolecules. A further increase in PAA concentration in the coagulation bath to 1.5 wt.% was found to increase the atomic concentration of oxygen up to 20.5% (Table 3).

The immobilization of PAA macromolecules to the membrane selective layer for SA-0.7 and SA-1.5 membranes was confirmed by the increased intensity of C=O bonds (at binding energy of 287.5 eV) compared to the SA-0 membrane (Figure 2) [41]. Moreover, the addition of PAA to the coagulation bath increased the C-O-C content, which is due to decreasing the degree of the washing out of Synperonic F108 during NIPS from the membrane matrix. This may be attributed to the formation of the Synperonic F108-PAA interpolymer complex [36,37,38].

### 3.2. The Effect of PAA Content in the Coagulation Bath on Membrane Structure

**Figure 3:**
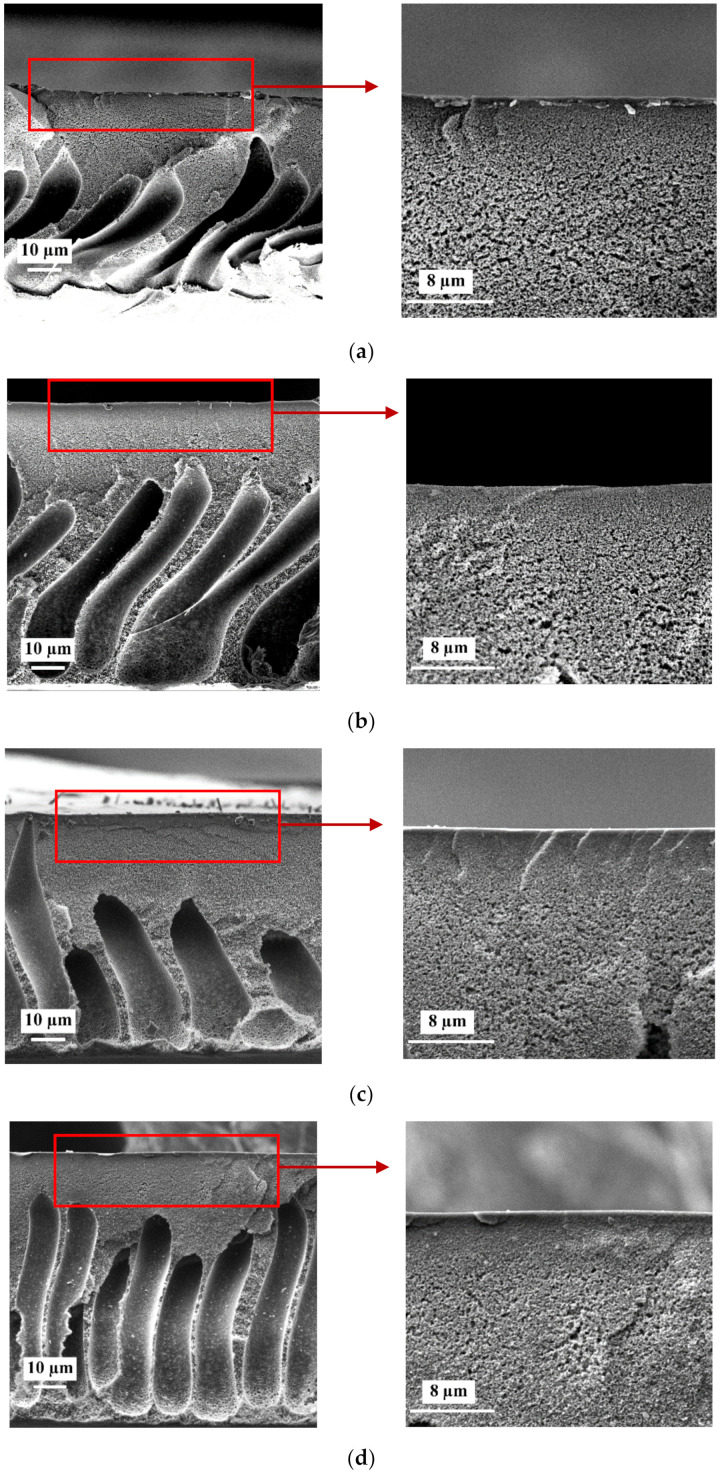

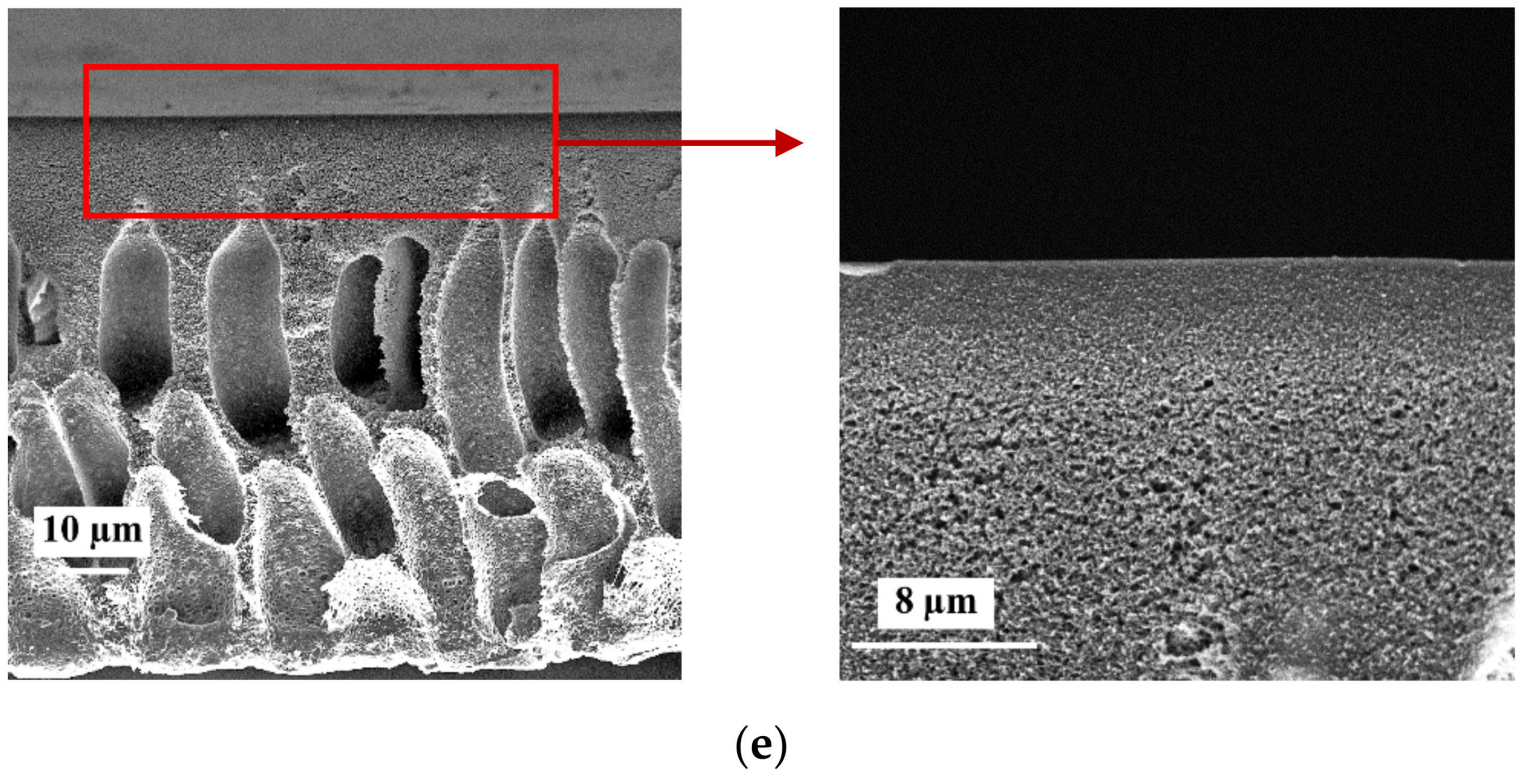
SEM microphotographs of the cross-section of the reference and modified membranes and enlarged fragment of cross-section in the vicinity of the selective layer: (**a**)—SA-0; (**b**)—SA-0.7; (**c**)—SA-1.0; (**d**)—SA-1.5; (**e**)—SA-2.0.

**Figure 4:**
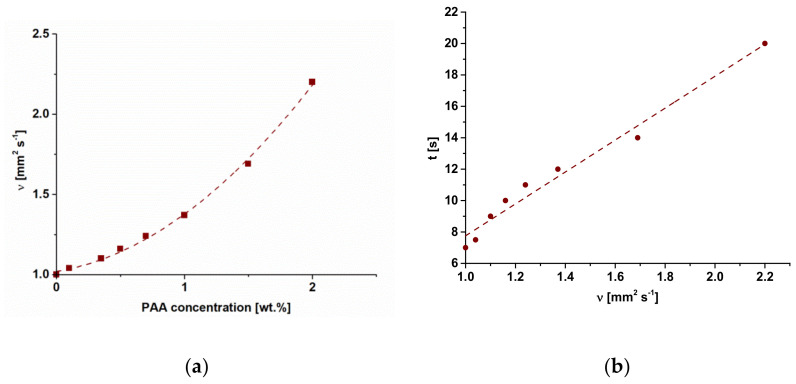
Dependence of kinematic viscosity (ν) of PAA aqueous solution on PAA concentration (**a**) and dependence of membrane formation time (t) on kinematic viscosity of PAA aqueous solution (**b**).

The microphotographs obtained by scanning electron microscopy studies of the reference PSF/Synperonic F108 and modified PSF/Synperonic F108/PAA membranes are presented in Figure 3. It was found that the reference and modified membranes feature a typical anisotropic structure formed by NIPS including a thin selective layer, transition sublayer, and porous sponge-like membrane matrix with large macrovoids. When PAA is added to the coagulation bath, the structure of membrane cross-section practically does not change. However, the selective layer becomes denser, thicker, and less porous when the PAA concentration in the coagulation bath increases. This can be due to the increase of viscosity of the coagulant when the PAA concentration increases, which results in the decrease of the “solvent-non-solvent” exchange rate in the NIPS process. The dependence of the viscosity of PAA aqueous solutions on the PAA concentration is presented in Figure 4a. It was found that an increase in PAA concentration yields a slight increase in the viscosity of the coagulant compared to water. For instance, an addition of 1.0 wt.% and 2.0 wt.% PAA results in a kinematic viscosity rise by 1.37 and 2.2 times compared to water. It was found in previous studies that the addition of the cationic and anionic polyelectrolytes based on polyacrylamide Praestol 859 and Praestol 2540 to the coagulation bath does not change the thermodynamics of the phase separation of the casting solution in NIPS [19,20,21,22]. However, the significant increase in viscosity of the coagulant is due to the extremely high molecular weight of Praestol flocculants, resulting in the dramatic increase in membrane formation time and delayed demixing of the casing solution [19,20,21,22]. In the case of using PAA as an additive to the coagulation bath, the membrane formation time increases to a much lower degree compared to the addition of Praestol flocculants (Figure 4b). When 2.0 wt.% PAA is added to the coagulant membrane, formation time rises by c.a. 2.4 times compared to using water as a coagulant (from 7 to 17 s) (Figure 4b).

It was shown that introduction of PAA to the coagulant results in only a little increase in membrane formation time since the PAA molecular weight is not very high (Figure 4b). It is worth noting that the membrane formation time is just an integral characteristic of the kinetic processes in NIPS and does not reveal the mechanism of the membrane structure formation. It is likely that a thicker and denser selective layer is formed because of the immobilization of PAA macromolecules in the selective layer and formation of hydrogen bonds with Synperonic F108. When the cast polymer film contacts the non-solvent in NIPS, a coagulation of the membrane-forming polymer (PSF) occurs, and a gelatinous, partly coagulated thin selective layer is formed. The further diffusion of solvent (DMAc) out of the nascent polymer film and non-solvent (water) and PAA macromolecules inside the polymer film occurs through this barrier layer, which demonstrates a sieving effect. Moreover, the segregation of Synperonic F108 occurs, which results in the enrichment of the surface of the selective layer with PEG blocks. The diffusion of polyelectrolyte chains through the yielded selective layer must be lower in comparison with the diffusion of solvent and non-solvent due to a higher molecular weight. It is supposed that the PAA will form hydrogen bonds with PEG blocks of Synperonic F108. PAA macromolecules are supposed to be located near the vicinity of the selective layer. The concentration of PAA macromolecules is expected to decrease sharply across the membrane thickness. FTIR studies have revealed no PAA macromolecules on the bottom layer of the modified membranes (Figure 1).

**Figure 5:**
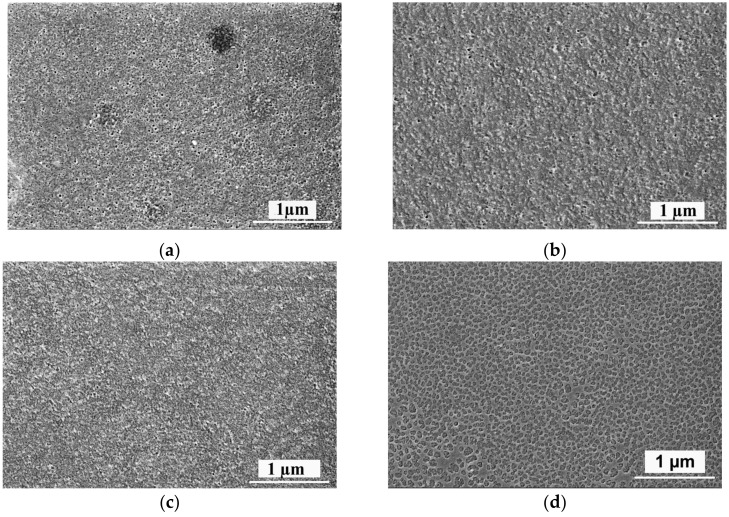

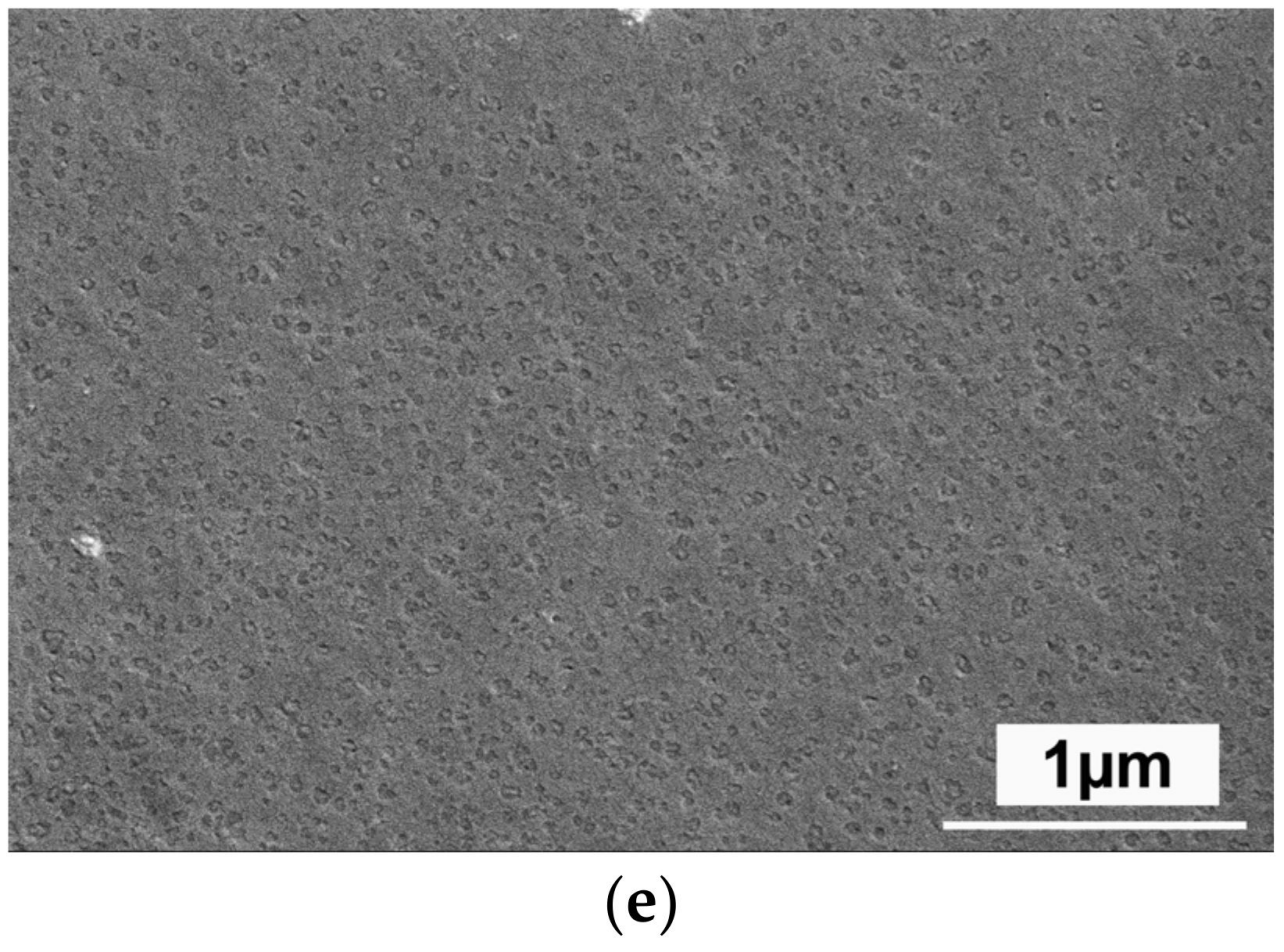
SEM microphotographs of the membrane selective layer surface: (**a**)—SA-0; (**b**)—SA-0.35; (**c**)—SA-0.7; (**d**)—SA-1.0; (**e**)—SA-2.0. Membrane samples were prepared by freeze-drying.

The presence of PAA macromolecules in the membrane structure resulted in decreasing pore size in the membrane selective layer from 17–27 nm for the SA-0 membrane to 7–20 and 7–17 nm for the SA-0.35 and SA-0.7 membranes, respectively (Figure 5a–c). This is due to the reduction of the phase inversion rate attributed to an increase in the viscosity of the coagulant (Figure 4). For SA-1.0 and SA-2.0 domains, materials different from the bulk surface of the selective layer are detected (Figure 5d,e). This could be due to the formation of an interpolymer complex of PAA and PEG blocks of Synperonic F108 (Figure 5d,e). In situ formation of the additional separation layer on the surface of PVDF ultrafiltration membranes when PVP was added to the casting solution and PAA was introduced in coagulation bath was revealed and proved by He et al. [23]. Formation of the layer of the PAA-Synperonic F108 interpolymer complex which partly covers the selective layer surface could prevent the detection of the pores by SEM for SA-1.0 and SA-2.0 membranes (Figure 5d,e). SEM images of the surface of the selective layer of membrane samples prepared via stepwise solvent exchange are presented in Figure S1 (Supplementary Materials). It is worth noting that formation of domains from other materials is clearly observed for SA-1.0 and SA-2.0 membranes (Figure S1, Supplementary Materials). Thus, regardless of the sample preparation method, stable domains from material other than the bulk membrane are observed on the surface of the selective layer, which confirms that it is not an effect of an impregnating agent or impurities.

### 3.3. AFM Studies of the Selective Layer Surface

**Figure 6:**
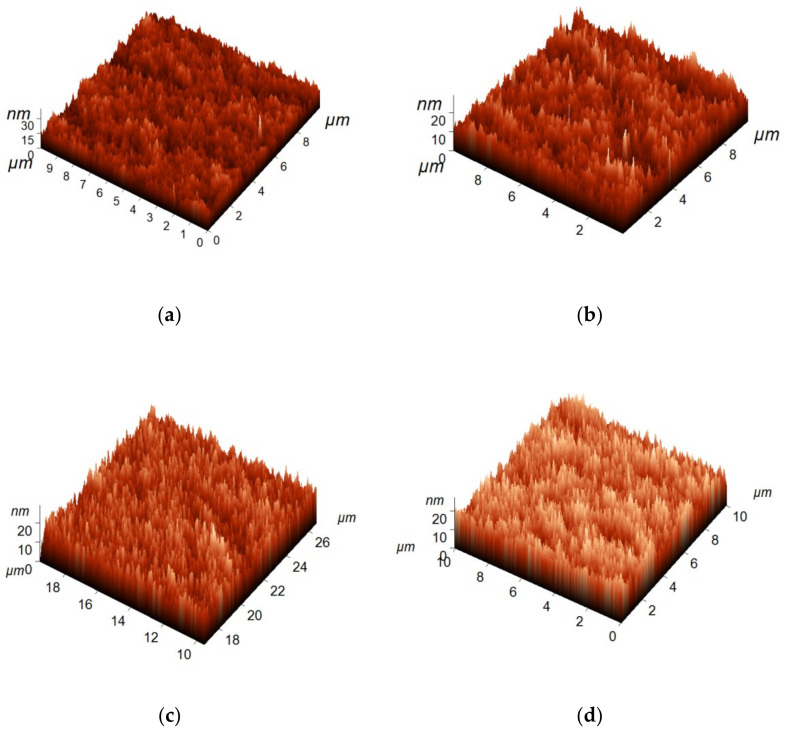
AFM micrographs of the surface of the selective layer of reference PSF/Synperonic F108 and PSF/Synperonic F108/PAA membranes: (**a**)—SA-0; (**b**)—SA-1.0; (**c**)—SA-1.5; (**d**)—SA-2.0.

The topology and surface roughness parameters of the membrane selective layer surface were studied using AFM (Figure 6).

The selective layer of modified membranes was found to have a nodular structure typical of membranes obtained via NIPS (Figure 6). Immobilization of the polyelectrolyte chains on the surface of the selective layer was shown to slightly influence the topology and selective layer roughness (Table 4). Average roughness (R_a_, nm) and root mean square roughness (R_q_, nm) parameters were found to slightly decrease when PAA was introduced to the coagulation bath. Moreover, roughness parameters were practically similar for all modified membranes. The lower surface roughness of PSF/Synperonic F108/PAA membranes compared to the reference membranes may be due to the pore size decrease and formation of the soft layer of the interpolymer complex of PEG blocks of Synperonic F108 and PAA. Based on the fact that PSF/Synperonic F108/PAA membranes demonstrate lower roughness parameters compared to the reference SA-0 membrane, it could be expected that modified membranes would demonstrate better antifouling stability compared to SA-0.

### 3.4. Measurements of Zeta Potential of the Membrane Surface

**Figure 7:**
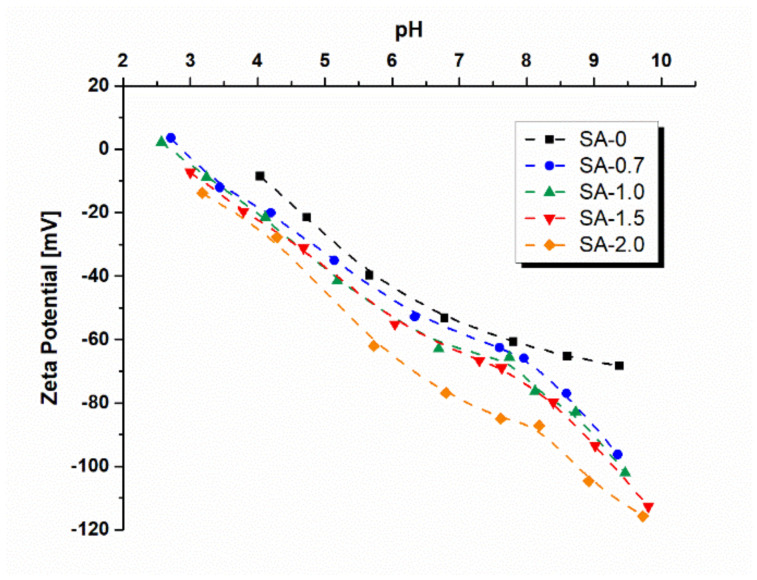
Dependence of zeta potential of the selective layer surface on pH of the reference PSF/Synperonic F108 (SA-0) and modified PSF/Synperonic F108/PAA (SA-0.7, SA-1.0, SA-1.5, and SA-2.0) membranes.

The PSF/Synperonic F108 and PSF/Synperonic F108/PAA membrane surface zeta potential was studied via measurements of the tangential flow streaming potential. Zeta potential is considered to be an important characteristic which significantly influences membrane separation and antifouling performance. Zero fit demonstrates that the isoelectric point for SA-0 membranes is approximately at pH 3.4 for SA-0.7–2.4, SA-1.0–2.3, SA-1.5, and SA-2.0 at 2.2 (Figure 7). Thus, an isoelectric point was found to significantly decrease with the increase in the PAA concentration. It is worth mentioning that the mechanism of the decrease in zeta potential of the selective layer surface of the SA-0 membrane when pH increases lies in the specific ionic adsorption of anions of electrolyte (Cl^−^) and hydroxide ions (OH^-^) since the membrane-forming polymer (PSF) and Synperonic F108 possess no dissociated groups [42]. It was shown that the reference PSF/Synperonic F108 (SA-0) membrane is characterized by a lower absolute value zeta potential compared to the PAA-modified membranes over the entire studied pH range. It was revealed that when PAA concentration in the coagulation bath increases, the surface charge of the membrane selective layer decreases (absolute value increases) because of the increase in the number of immobilized PAA macromolecules, which are capable of ionization (pKa of PAA is 4.5 [43]) (Figure 2, Figure 3, and Figure 7, Table 3).

### 3.5. Hydropilicity of the Membrane Selective Layer

**Figure 8:**
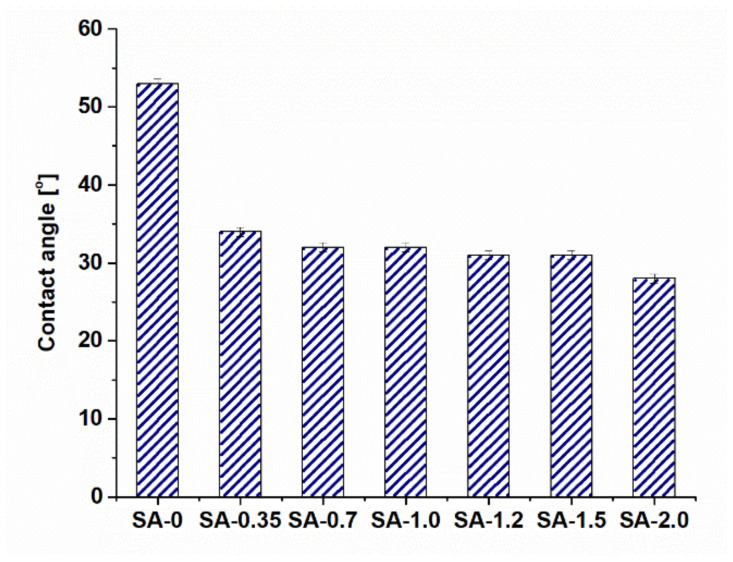
Correlation between water CA of the selective layer and PAA concentration in the coagulation bath.

Membrane surface hydrophilicity was investigated by determination of the water CA of the membrane surface by the attached air bubble technique. Hydrophilic–hydrophobic balance is known to determine the resistance of membranes to fouling [33]. The contact angle of the PSF-0 membrane containing only the membrane-forming polymer without any additives and prepared using water as a coagulant was determined to be 65°. It is worth noting that the reference PSF/Synperonic F108 membrane features a lower water contact angle of 53° compared to the contact angle of the PSF-0 membrane. This is due to the presence of hydrophilic PEG blocks of Synperonic F108 on the surface of the membrane selective layer [44]. It was shown that the addition of PAA into the coagulation bath during membrane preparation resulted in a significant decrease in water CA from 53° to 34° for the reference SA-0 and SA-0.35 membranes, correspondingly (Figure 8). A further increase in PAA concentration has shown to decrease CA down to 31–32° for modified membranes SA-0.7, SA-1.0, and SA-1.5. It was revealed that the SA-2.0 membrane is characterized by the most hydrophilic selective layer surface (CA is 28°) due to the highest amount of immobilized PAA macromolecules into the membrane matrix (Figure 8). It is supposed that carboxylic groups of PAA could form hydrogen bonds with PEG chains of Synperonic F108 on the surface of the membrane selective layer, which can result in the formation of the interpolymer complex Synperonic F108/PAA (Figure 5,d,e). The highly hydrophilic interpolymer complex Synperonic F108/PAA could also be solvated by water molecules. This leads to formation of the hydrated layer, which prevents the absorption of organic and colloidal foulants on the membrane surface, increasing membrane fouling resistance [23,44]. It is widely known that contact angle depends both on the composition and roughness. A decrease in the CA of the membrane surface when PAA is added to the coagulation bath is consistent with the decrease in surface roughness parameters (Table 4, Figure 8). However, it is the change of composition that mainly contributes to the hydrophilization of the membrane selective layer (Figure 1 and Figure 2, Table 3).

### 3.6. Membrane Performance in Ultrafiltration of Model Solutions

**Figure 9:**
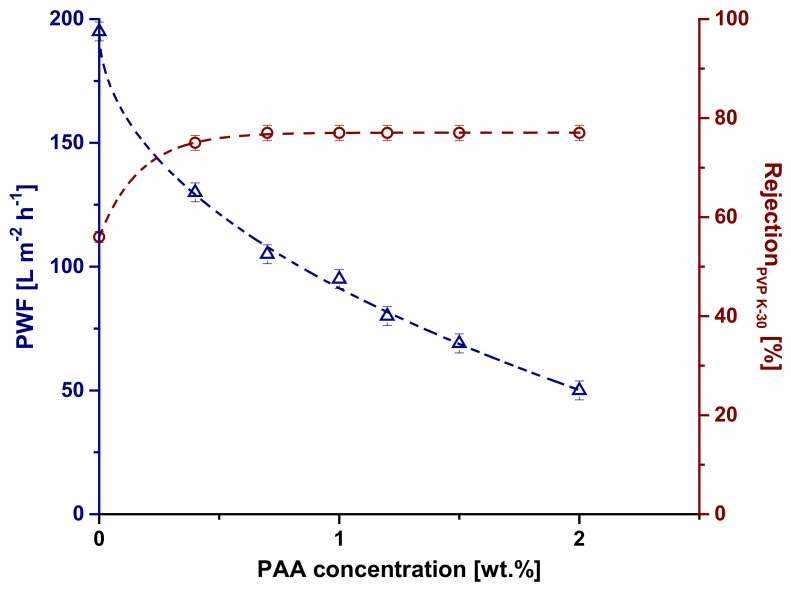
Dependence of PWF and PVP K-30 rejection coefficient (Rejection_PVP K-30_) on PAA concentration in the coagulation bath.

Selective layer thickness, pore size, and porosity primarily define membrane transport properties in ultrafiltration. It was found that membrane pure water flux (PWF) substantially decreases with an increase in PAA concentration. It was shown that the PWF for the SA-2.0 membrane decreased by four times compared to the reference SA-0 membrane (Figure 9) due to the formation of a denser selective layer and the decreasing pore size and porosity of the membrane selective layer (Figure 5).

It was found that introduction of PAA to the coagulation bath results in a major increase in PVP K-30 rejection (Figure 9). The reference PSF/Synperonic F108 membrane demonstrates 56% rejection of PVP K-30. The introduction of 0.35 wt.% PAA to the coagulation bath yields in a rise in PVP K-30 rejection up to 75% (Figure 9). It was revealed that the rejection increases up to 75 wt.% for the SA-0.7 membrane and does not change with a further increase in PAA concentration. The increase in PVP K-30 rejection is due to the decrease in membrane pore size.

**Figure 10:**
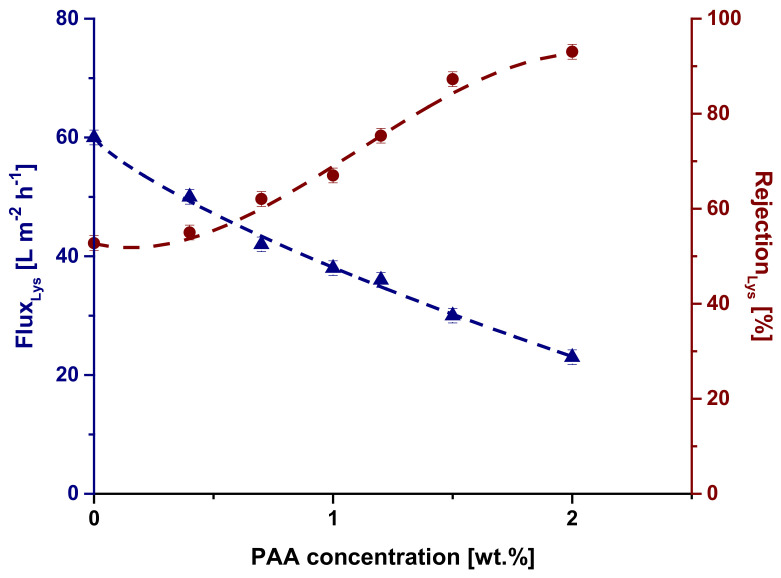
The dependence of the lysozyme solution flux (Flux_Lys_) and lysozyme rejection coefficient (Rejection_Lys_) on PAA concentration.

It was found that lysozyme solution flux monotonically decreased with an increase in PAA concentration in the coagulation bath (Figure 10). For instance, lysozyme solution flux decreased 2.6 times from 60 L m^−2^ h^−1^ for SA-0 to 23 L m^−2^ h^−1^ for the SA-2.0 membrane. It was shown that lysozyme rejection increases with an increase in PAA concentration. Lysozyme rejection increased from 55% for the reference membrane to 93% for SA-2.0. The decrease in lysozyme solution flux and increase in rejection are attributed to the pore size decrease (Figure 5) as well as the electrostatic attraction of the negatively charged membrane selective layer surface (Figure 7) and positively charged lysozyme macromolecules at pH 7.2. Electrostatic attraction may result in the formation of a lysozyme gel layer and thus the increase in lysozyme rejection. Moreover, the increase in lysozyme rejection can be attributed to the formation of the Synperonic F108/PAA interpolymer complex on the membrane selective layer surface, which partially blocks the pores.

### 3.7. The Effect of PAA Concentration on Membrane Stability to Fouling

#### 3.7.1. Ultrafiltration of HAs Solution

The antifouling performance of SA-0 and modified SA-1.5 membranes was studied and compared. The results of HAs solution ultrafiltration are presented in Table 5. The HAs solution flux of the SA-0 membrane was found to decrease from 204 to 168 L m^−2^ h^−1^ in an hour at a transmembrane pressure of 2 bar. Nevertheless, the HAs flux decline of the SA-1.5 membrane was significantly lower—from 168 to 150 L m^−2^ h^−1^. It was revealed that the PWF of the SA-0 membrane just after ultrafiltration of the HAs solution was 312 L m^−2^ h^−1^, compared to the initial PWF equal to 420 L m^−2^ h^−1^ before HAs solution ultrafiltration. The PWF of SA-1.5 before HAs filtration was 150 L m^−2^ h^−1^, and just after ultrafiltration, it was 138 L m^−2^ h^−1^. The membrane PWF after the ultrafiltration of MilliQ water for 30 min for SA-0 was found to increase to 336 L m^−2^ h^−1^, and for the SA-1.5 membrane, up to 144 L m^−2^ h^−1^. Based on these data, antifouling parameters were calculated. It was shown that the modified membrane SA-1.5 demonstrates a much higher FRR (92%) in comparison to the reference one (74%) (Table 5), probably due to the smaller pore size of PAA-modified membrane (Figure 4a,b), smoother surface (Table 4), significantly higher hydrophilicity (Figure 8), and more negatively charged selective layer surface (Figure 7). It is known that HAs molecules possess a negative charge at pH 8.4 [45], and thus the electrostatic repulsion of HAs molecules from the selective layer surface with a higher absolute value of negative charge of the SA-1.5 membrane (Figure 7) causes the higher FRR of the modified membranes.

The permeate characteristics of HAs model solution ultrafiltration are presented in Table 5.

It was revealed that the modified SA-1.5 membrane demonstrates better removal of color and iron compared to the reference SA-0 membrane. The higher degree of color removal is attributed to the smaller pore size of the selective layer as well as the presence of the interpolymer complex Synperonic F108/PAA on the membrane surface. The higher iron removal may be attributed to the complexation of iron ions with carboxylic groups of HAs and better rejection of these complexes by the SA-1.5 membrane with a smaller pore size of the selective layer in comparison with the SA-0 membrane (Figure 5a,d).

#### 3.7.2. Ultrafiltration of the ThMP Process Water

For ThMP process water filtration, SA-0.35, SA-0.5, and SA-0.7 modified membranes were selected for comparison with the reference SA-0 membrane due to their relatively high PWF compared to the membranes with a higher PAA concentration in the coagulation bath, while CA, zeta potential and surface roughness parameters of the membrane selective layer surface did not significantly change with an increase in PAA concentration.

**Figure 11:**
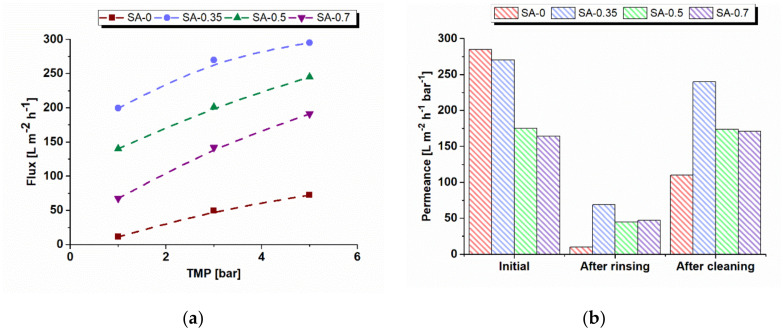
(**a**) Dependence of flux of ThMP process water of the reference and modified membranes on transmembrane pressure (TMP); (**b**) changes in pure water permeance correlated to the PAA concentration and chemical cleaning results for reference and modified membranes.

The ThMP process water flux at different transmembrane pressures and pure water permeances for PSF/Synperonic F108 and PSF/Synperonic F108/PAA membranes is demonstrated in Figure 11.

It was revealed that the membrane flux of ThMP process water increased non-monotonically with a higher concentration of polyelectrolytes in the coagulation bath, with the maximum for the SA-0.35 membrane. The rise of ThMP process water fluxes can be related to a higher antifouling stability of modified membranes because of the significant increase of hydrophilicity, slight decrease in surface roughness, and increase in absolute value of the negative charge of the membrane selective layer. These factors reduce the attachment of organic and colloid substances on the membrane surface, which can block the pores and cause the decline of permeability. On the other hand, it was previously found that PWF decreases when PAA concentration in the coagulation bath increases (Figure 9). This tendency is attributed to the decrease in the pore size and porosity of the membrane selective layer and increase in the selective layer thickness (Figure 3, Figure 5, and Figure 9). It can be concluded that pore size, porosity, and thickness of the selective layer have a higher impact on the membrane flux compared to hydrophilicity [46]. The maximum ThMP process water flux for the SA-0.35 membrane can be explained by the following: the pore size of the selective layer has declined to a lower extent compared to other modified membranes, while hydrophilicity is comparable to the other modified membranes (Figure 5 and Figure 8).

Membrane permeance using deionized water was measured prior to ThMP process water ultrafiltration, after deionized water rinsing and after chemical cleaning with an alkaline agent after the ultrafiltration of ThMP process water (Figure 11b). Initial pure water permeance of the modified membranes was found to be lower compared to the permeance of the reference membrane. This is the result of the decrease in pore size and porosity of the membrane selective layer and increase in its thickness (Figure 3 and Figure 5). However, the permeance of PAA-modified membranes after deionized water rinsing turned out to be much higher compared to the SA-0 membrane. Furthermore, it was determined to be 6.9 times higher in the case of the SA-0.35 membrane compared to the reference membrane, and 4.5 and 4.7 times higher for SA-0.5 and SA-0.7 membranes (Figure 11b). Permeance was demonstrated to increase up to 11 times after the membrane cleaning for SA-0 and by 3.6–3.9 times for PAA-modified membranes compared to the permeance after deionized water rinsing. Nonetheless, membrane permeance after deionized water rinsing and alkaline cleaning did not surpass the initial permeance, which reveals the occurrence of irreversible membrane fouling.

**Figure 12:**
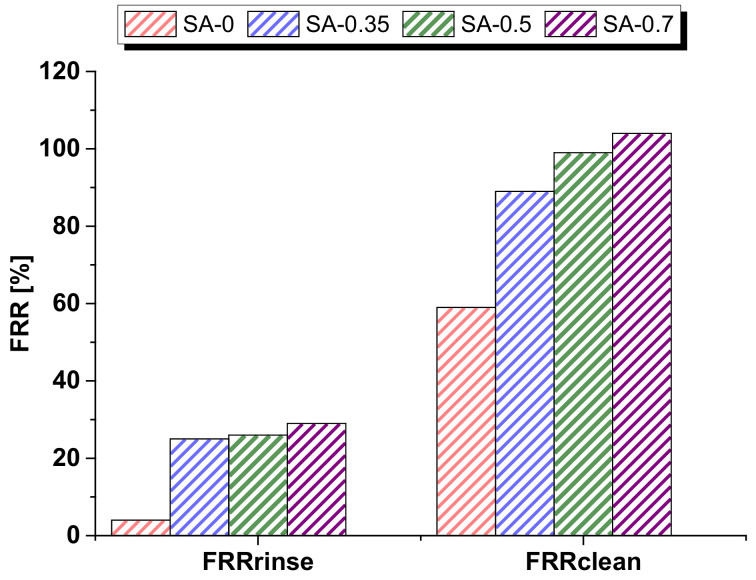
Dependence of FRR of the reference SA-0 and PAA-modified membranes after ThMP process water ultrafiltration on the cleaning technique: deionized water rinsing (FRR_rinse_) and alkaline chemical cleaning (FRR_clean_).

According to the results obtained and presented in Figure 12, membrane modification by PAA reduced the degree of irreversible fouling, especially in the case of membranes prepared with 0.5 and 0.7 wt.% PAA in the coagulation bath. It was revealed that higher hydrophilicity and the more negatively charged selective layer surface of the modified membranes yield the improvement of the ThMP process water flux as well as decrease membrane fouling due to reducing the number of interactions between the membrane surface and dissolved substances in the feed solution. For instance, after cleaning with Ultrasil 10, FRR is 59% for the reference PSF/Synperonic F108 (SA-0) membrane and 99% and 104% for the SA-0.5 and SA-0.7 membranes, respectively (Figure 12).

#### 3.7.3. Total Lignin and Hemicellulose Rejection

**Figure 13:**
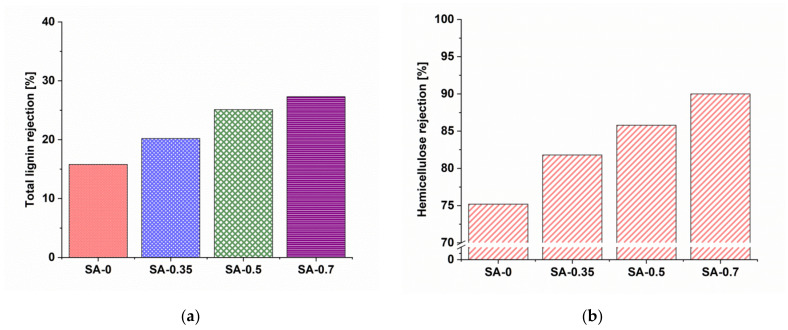
Total lignin (**a**) and hemicellulose (**b**) rejection of the reference PSF/Synperonic F108 and modified PSF/Synperonic F108/PAA membranes in ultrafiltration of ThMP process water.

It was found that the increase in PAA concentration in the coagulation bath during membrane formation increases both the rejection of lignin and hemicellulose (Figure 13). The reference PSF/Synperonic F108 membrane retained 15.8% of total lignin and 75.2% of hemicellulose. The SA-0.7 membrane demonstrated 27.3% rejection of total lignin and 90% rejection of hemicellulose.

The modified membranes demonstrated more efficient concentrating performance of hemicellulose. This is correlated to the smaller pore size of the selective layer compared to the SA-0 membrane. This also means that the overall product yield increases, as less hemicellulose is eluted when the rejection is higher. The difference in rejections between hemicellulose and lignin is slightly higher for the SA-0.7 membrane (62.7%) compared to the SA-0 membrane (59.4%). This provides the possibility of purifying hemicellulose in ThMP process water treatment in pulping industries.

The comparison of the flux and hemicelluloses and lignin rejection in ThMP process water ultrafiltration of the developed membranes with commercially available membranes ETNA10, ETNA01, and UFX5 (Alfa Laval Nakskov A/S, Nakskov, Denmark) is presented in Table 6. Note that the conditions of ultrafiltration reported in the literature were different, so the comparison is not strict. It was found that the SA-0.35, SA-0.5, and SA-0.7 membranes demonstrated a combination of high flux and quite high rejection. The SA-0.7 membrane significantly outperforms the ETNA01 membrane by flux and hemicellulose rejection.

However, the lignin rejection is lower for the SA-0.7 (27%) membrane compared to ETNA01 (34%). The SA-0.35 membrane demonstrates better flux and higher rejection compared to the ETNA10 membrane; however, the transmembrane pressure and feed solution temperature are lower in the case of the ETNA10 membrane. All developed membranes were found to have a lower rejection of hemicelluloses and lignin compared to the UFX5 membrane, but higher flux. Overall, the developed membranes are characterized by higher flux and a high level of rejection compared to the commercial membranes (Table 6).

## 4. Conclusions

A systematic study of the method of modification of PSF/Synperonic F108 ultrafiltration membranes with polyacrylic acid (PAA) as an additive to the coagulation bath during NIPS was carried out. It was revealed that the addition of PAA into the coagulation bath leads to the formation of a denser selective layer with a smaller pore size and lower porosity. This causes a decrease in the membrane pure water and lysozyme solution flux as well as an increase in PVP K-30 and lysozyme rejection. The membrane surface was found to become more hydrophilic and negatively charged with an increase in PAA concentration due to the immobilization of PAA macromolecules on the membrane selective layer surface as well as on the pore walls, which is facilitated by the formation of an interpolymer complex with the triblock copolymer Synperionic F108.

PAA-modified membranes demonstrated better antifouling stability and higher color and iron removal in the ultrafiltration of HAs compared to the reference membrane. In ThMP process water ultrafiltration, modified membranes achieved higher flux, FRR, and total lignin and hemicellulose rejection compared to the reference membrane. The difference of rejection coefficients of hemicelluloses and total lignin of modified membranes indicates the possibility for hemicellulose purification and simultaneously reaching high process yields. This makes the modified membranes suitable for the recovery of hemicelluloses from the ThMP process water in the pulp industry.

## Figures and Tables

**Table 1:** Designations of PSF/Synperonic F108 membranes prepared using different PAA concentrations in the coagulation bath.

Designation	PAA Concentration in the Coagulation Bath [wt.%]
SA-0	0
SA-0.35	0.35
SA-0.5	0.5
SA-0.7	0.7
SA-1.0	1.0
SA-1.2	1.2
SA-1.5	1.5
SA-2.0	2.0

**Table 2:** Parameters of the HAs solution in tap water.

Parameter	Value
Color (λ = 400 nm)	0.50
pH	8.4
c (Fe) [mg L^−1^]	257

**Table 3:** Elemental analysis of the surface of the selective layer of pristine PSF membrane, PSF/Synperonic F108, and PSF/Synperonic F108/PAA membranes.

Membrane Abbreviation	Composition of Selective Layer Surface, Atomic Percent Concentration (%)			
	C	S	O	N
PSF-0	83.3	3.5	12.3	0.9
SA-0	82.1	2.7	14	1.2
SA-0.7	77.9	2.4	18.5	-
SA-1.5	76.6	1.9	20.5	1.1

**Table 4:** The surface roughness parameters of the reference PSF/Synperonic F108 and PSF/Synperonic F108/PAA membranes.

Membrane Designation	Roughness Parameters	
	R_a_ [nm]	R_q_ [nm]
SA-0	2.9	3.5
SA-1.0	2.1	2.8
SA-1.5	2.6	3.2
SA-2.0	2.6	3.2

**Table 5:** Membrane performance and permeate parameters of reference PSF/Synperonic F108 and modified PSF/Synperonic F108/PAA membranes.

Membrane Designation	J_Has_ [L m^−2^ h^−1^]	FRR [%]	DT [%]	Permeate Parameters		
				Color (λ = 400 nm)	pH	c (Fe) [mg L^−1^]
SA-0	168	74	20	0.11	8.4	0.15
SA-1.5	150	92	0	0.01	8.2	0.05

**Table 6:** Comparison of membrane performance in ThMP process water ultrafiltration at concentration of hemicellulose 2 g·L^−1^.

Membrane Code	UF Conditions		Flux, L·m^−2^·h^−1^	Rejection, %		Reference
	P, bar	T, °C		Hemicellulose	Lignin	
ENTA10	2.5	60	160	76	15	[4]
ETNA01	4	60	40	87	34	[4]
UFX5	4	75	120	98	32	[4]
SA-0	3	70	49	75	16	This study
SA-0.35	3	70	270	82	20	This study
SA-0.5	3	70	201	83	25	This study
SA-0.7	3	70	142	90	27	This study

## Data Availability

The data presented in this study are available on request from the corresponding author. The data are not publicly available due to being a part of ongoing research.

## Supplementary Materials

The following supporting information can be downloaded at: https://www.mdpi.com/article/10.3390/ma15010359/s1, Figure S1: SEM microphotographs of the membrane selective layer surface: a—SA-0; b—SA-0.35; c—SA-0.7; d—SA-1.0; e—SA-2.0. Membrane samples were prepared by stepwise solvent exchange.

## References

[1] Singh A.K., Chandra R. (2019). Pollutants released from the pulp paper industry: Aquatic toxicity and their health hazards. Aquat. Toxicol..

[2] Persson T., Krawczyk H., Nordin A.-K., Jönsson A.-S. (2010). Fractionation of process water in thermomechanical pulp mills. Bioresour. Technol..

[3] Krawczyk H., Jönsson A.-S. (2011). Separation of dispersed substances and galactoglucomannan in thermomechanical pulp process water by microfiltration. Sep. Purif. Technol..

[4] Persson T., Jönsson A.-S. (2010). Isolation of hemicelluloses by ultrafiltration of thermomechanical pulp mill process water—Influence of operating conditions. Chem. Eng. Res. Des..

[5] Alkhouzaam A., Qiblawey H. (2021). Novel polysulfone ultrafiltration membranes incorporating polydopamine functionalized graphene oxide with enhanced flux and fouling resistance. J. Membr. Sci..

[6] Jönsson A.-S. (2016). Membranes for lignin and hemicellulose recovery in pulp mills. Membrane Technologies for Biorefining.

[7] Zhao C., Song T., Yu Y., Qu L., Cheng J., Zhu W., Wang Q., Li P., Tang W. (2020). Insight into the influence of humic acid and sodium alginate fractions on membrane fouling in coagulation-ultrafiltration combined system. Environ. Res..

[8] Shokri E., Shahed E., Hermani M., Etemadi H. (2021). Towards enhanced fouling resistance of PVC ultrafiltration membrane using modified montmorillonite with folic acid. Appl. Clay Sci..

[9] Lu X., Peng Y., Qiu H., Liu X., Ge L. (2017). Anti-fouling membranes by manipulating surface wettability and their anti-fouling mechanism. Desalination.

[10] Bhalani D.V., Trivedi J.S., Jewrajka S.K. (2021). Selective grafting of morphologically modified poly(vinylidene fluoride) ultrafiltration membrane by poly(acrylic acid) for inducing antifouling property. Appl. Surf. Sci..

[11] Bildyukevich A.V., Plisko T.V., Isaichykova Y.A., Ovcharova A.A. (2018). Preparation of High-Flux Ultrafiltration Polyphenylsulfone Membranes. Pet. Chem..

[12] Plisko T.V., Bildyukevich A.V., Usosky V.V., Volkov V.V. (2016). Influence of the concentration and molecular weight of polyethylene glycol on the structure and permeability of polysulfone hollow fiber membranes. Pet. Chem..

[13] Chakrabarty B., Ghoshal A., Purkait M. (2008). SEM analysis and gas permeability test to characterize polysulfone membrane prepared with polyethylene glycol as additive. J. Colloid Interface Sci..

[14] Ohya H., Shiki S., Kawakami H. (2009). Fabrication study of polysulfone hollow-fiber microfiltration membranes: Optimal dope viscosity for nucleation and growth. J. Membr. Sci..

[15] Chakrabarty B., Ghoshal A., Purkait M. (2008). Effect of molecular weight of PEG on membrane morphology and transport properties. J. Membr. Sci..

[16] Plisko T.V., Bildyukevich A.V., Karslyan Y.A., Ovcharova A.A., Volkov V.V. (2018). Development of high flux ultrafiltration polyphenylsulfone membranes applying the systems with upper and lower critical solution temperatures: Effect of polyethylene glycol molecular weight and coagulation bath temperature. J. Membr. Sci..

[17] Burts K.S., Plisko T.V., Bildyukevich A.V., Penkova A.V., Pratsenko S.A. (2021). Modification of polysulfone ultrafiltration membranes using block copolymer Pluronic F127. Polym. Bull..

[18] Plisko T., Penkova A., Burts K., Bildyukevich A., Dmitrenko M., Melnikova G., Atta R., Mazur A., Zolotarev A., Missyul A. (2019). Effect of Pluronic F127 on porous and dense membrane structure formation via non-solvent induced and evaporation induced phase separation. J. Membr. Sci..

[19] Plisko T.V., Bildyukevich A.V., Burts K.S., Ermakov S.S., Penkova A.V., Kuzminova A.I., Dmitrenko M.E., Hliavitskaya T.A., Ulbricht M. (2020). One-Step Preparation of Antifouling Polysulfone Ultrafiltration Membranes via Modification by a Cationic Polyelectrolyte Based on Polyacrylamide. Polymers.

[20] Plisko T.V., Bildyukevich A.V., Burts K.S., Hliavitskaya T.A., Penkova A.V., Ermakov S.S., Ulbricht M. (2020). Modification of Polysulfone Ultrafiltration Membranes via Addition of Anionic Polyelectrolyte Based on Acrylamide and Sodium Acrylate to the Coagulation Bath to Improve Antifouling Performance in Water Treatment. Membranes.

[21] Hliavitskaya T., Plisko T., Bildyukevich A., Lipnizki F., Rodrigues G., Sjölin M. (2020). Modification of PES ultrafiltration membranes by cationic polyelectrolyte Praestol 859: Characterization, performance and application for purification of hemicellulose. Chem. Eng. Res. Des..

[22] Hliavitskaya T., Plisko T., Pratsenko S., Bildyukevich A., Lipnizki F., Rodrigues G., Sjölin M. (2021). Development of antifouling ultrafiltration PES membranes for concentration of hemicellulose. J. Appl. Polym. Sci..

[23] He M., Su Y., Zhang R., Liu Y., Zhang S., Jiang Z. (2018). In-situ construction of antifouling separation layer via a reaction enhanced surface segregation method. Chem. Eng. Sci..

[24] Liu C., Mao H., Zheng J., Zhang S. (2017). In situ surface crosslinked tight ultrafiltration membrane prepared by one-step chemical reaction-involved phase inversion process between activated PAEK-COOH and PEI. J. Membr. Sci..

[25] Burts K.S., Plisko T.V., Bildyukevich A.V., Rodrigues G., Sjölin M., Lipnizki F., Ulbricht M. (2021). Development of polysulfone ultrafiltration membranes with enhanced antifouling performance for the valorisation of side streams in the pulp and paper industry. Colloids Surf. A Physicochem. Eng. Asp..

[26] Al-Rudainy B. (2020). Galactoglucomannan Recovery from Softwood Spent Sulfite Liquor: Challenges, Process Design and Techno-Economic Evaluations. Ph.D. Thesis.

[27] Sluiter A., Hames B., Ruiz R., Scarlata C., Sluiter J., Templeton D. (2006). Determination of sugars, byproducts, and degradation products in liquid fraction process samples. Gold.: Natl. Renew. Energy Lab..

[28] Sluiter A., Hames B., Ruiz R., Scarlata C., Sluiter J., Templeton D., Crocker D.L. (2008). Determination of structural carbohydrates and lignin in biomass. Lab. Anal. Proced..

[29] Holmbom B., Thornton J. (1997). Dissolution and dispersion of spruce wood components into hot water. Wood Sci. Technol..

[30] Singh R., Sinha M.K., Purkait M.K. (2020). Stimuli responsive mixed matrix polysulfone ultrafiltration membrane for humic acid and photocatalytic dye removal applications. Sep. Purif. Technol..

[31] Farrokhara M., Dorosti F. (2020). New high permeable polysulfone/ionic liquid membrane for gas separation. Chin. J. Chem. Eng..

[32] Yu S., Zhu J., Liao J., Ruan H., Sotto A., Shen J. (2021). Homogeneous trimethylamine-quaternized polysulfone-based anion exchange membranes with crosslinked structure for electrodialysis desalination. Sep. Purif. Technol..

[33] Yunos M.Z., Harun Z., Basri H., Ismail A.F. (2014). Studies on fouling by natural organic matter (NOM) on polysulfone membranes: Effect of polyethylene glycol (PEG). Desalination.

[34] Susanto H., Ulbricht M. (2009). Characteristics, performance and stability of polyethersulfone ultrafiltration membranes prepared by phase separation method using different macromolecular additives. J. Membr. Sci..

[35] Silva L.L., Abdelraheem W., Nadagouda M.N., Rocco A.M., Dionysiou D.D., Fonseca F.V., Borges C.P. (2021). Novel microwave-driven synthesis of hydrophilic polyvinylidene fluoride/polyacrylic acid (PVDF/PAA) membranes and decoration with nano zero-valent-iron (nZVI) for water treatment applications. J. Membr. Sci..

[36] Costa T., de Melo S.S., Miguel M.D.G., Lindman B., Schillén K. (2009). Complex Formation between a Fluorescently-Labeled Polyelectrolyte and a Triblock Copolymer. J. Phys. Chem. B.

[37] Pragatheeswaran A.M., Chen S.B. (2016). The influence of poly(acrylic acid) on micellization and gelation characteristics of aqueous Pluronic F127 copolymer system. Colloid Polym. Sci..

[38] Seo J., Moon J., Moon S., Paik U. (2015). Interpolymer complexes of poly(acrylic acid) and poly(ethylene glycol) for low dishing in STI CMP. Appl. Surf. Sci..

[39] Zhu L.-J., Song H.-M., Wang G., Zeng Z.-X., Zhao C.-T., Xue Q.-J., Guo X.-P. (2018). Microstructures and performances of pegylated polysulfone membranes from an in situ synthesized solution via vapor induced phase separation approach. J. Colloid Interface Sci..

[40] Barambu N.U., Bilad M.R., Bustam M.A., Huda N., Jaafar J., Narkkun T., Faungnawakij K. (2020). Development of Polysulfone Membrane via Vapor-Induced Phase Separation for Oil/Water Emulsion Filtration. Polymers.

[41] Tu M.-M., Xu J.-J., Qiu Y.-R. (2019). Surface hemocompatible modification of polysulfone membrane via covalently grafting acrylic acid and sulfonated hydroxypropyl chitosan. RSC Adv..

[42] Möckel D., Staude E., Dal-Cin M., Darcovich K., Guiver M. (1998). Tangential flow streaming potential measurements: Hydrodynamic cell characterization and zeta potentials of carboxylated polysulfone membranes. J. Membr. Sci..

[43] Yi G., Fan X., Quan X., Zhang H., Chen S., Yu H. (2019). A pH-responsive PAA-grafted-CNT intercalated RGO membrane with steady separation efficiency for charged contaminants over a wide pH range. Sep. Purif. Technol..

[44] Elcik H., Cakmakci M., Özkaya B. (2017). Preparation and characterisation of novel polysulfone membranes modified with Pluronic F-127 for reducing microalgal fouling. Chem. Pap..

[45] Klučáková M. (2018). Conductometric study of the dissociation behavior of humic and fulvic acids. React. Funct. Polym..

[46] Rahimpour A., Madaeni S.S. (2010). Improvement of performance and surface properties of nano-porous polyethersulfone (PES) membrane using hydrophilic monomers as additives in the casting solution. J. Membr. Sci..

