# Supplementary material for: Development of Antifouling Polysulfone Membranes by Synergistic Modification with Two Different Additives in Casting Solution and Coagulation Bath: Synperonic F108 and Polyacrylic Acid
Source: Materials (Basel). 2022 Jan 4;15(1):359. doi: 10.3390/ma15010359 (PMC8746107; doi:10.3390/ma15010359)
Supplement: Supplementary file 1 [file materials-15-00359-s001.zip › materials-1452870-supplementary.pdf]

Supplementary materials

Development of Antifouling Polysulfone Membranes by Synergistic Modification with Two Different Additives in Casting Solution and Coagulation Bath: Synperonic F108 and Polyacrylic Acid

Katsiaryna S. Burts [1], Tatiana V. Plisko [1,*], Mikael Sjölin [2], Goncalo Rodrigues [2,3], Alexandr V. Bildyukevich [1], Frank Lipnizki [2] and Mathias Ulbricht [4]

[1] Institute of Physical Organic Chemistry, National Academy of Sciences of Belarus, 220072, Minsk, Belarus; katyaburt@gmail.com (K.S.B.); uf@ifoch.bas-net.by (A.V.B.)

[2] Department of Chemical Engineering, Lund University, 221 00, Lund, Sweden; mikael.sjolin@chemeng.lth.se (M.S.); goncaloncrodrigues@ist.utl.pt (G.R.); frank.lipnizki@chemeng.lth.se (F.L.)

[3] Department of Bioengineering, Instituto Superior Técnico, 1049-001, Lisbon, Portugal

[4] Lehrstuhl für Technische Chemie II, Universität Duisburg-Essen, 45141, Essen, Germany; mathias.ulbricht@uni-essen.de

* Correspondence: plisko.v.tatiana@gmail.com

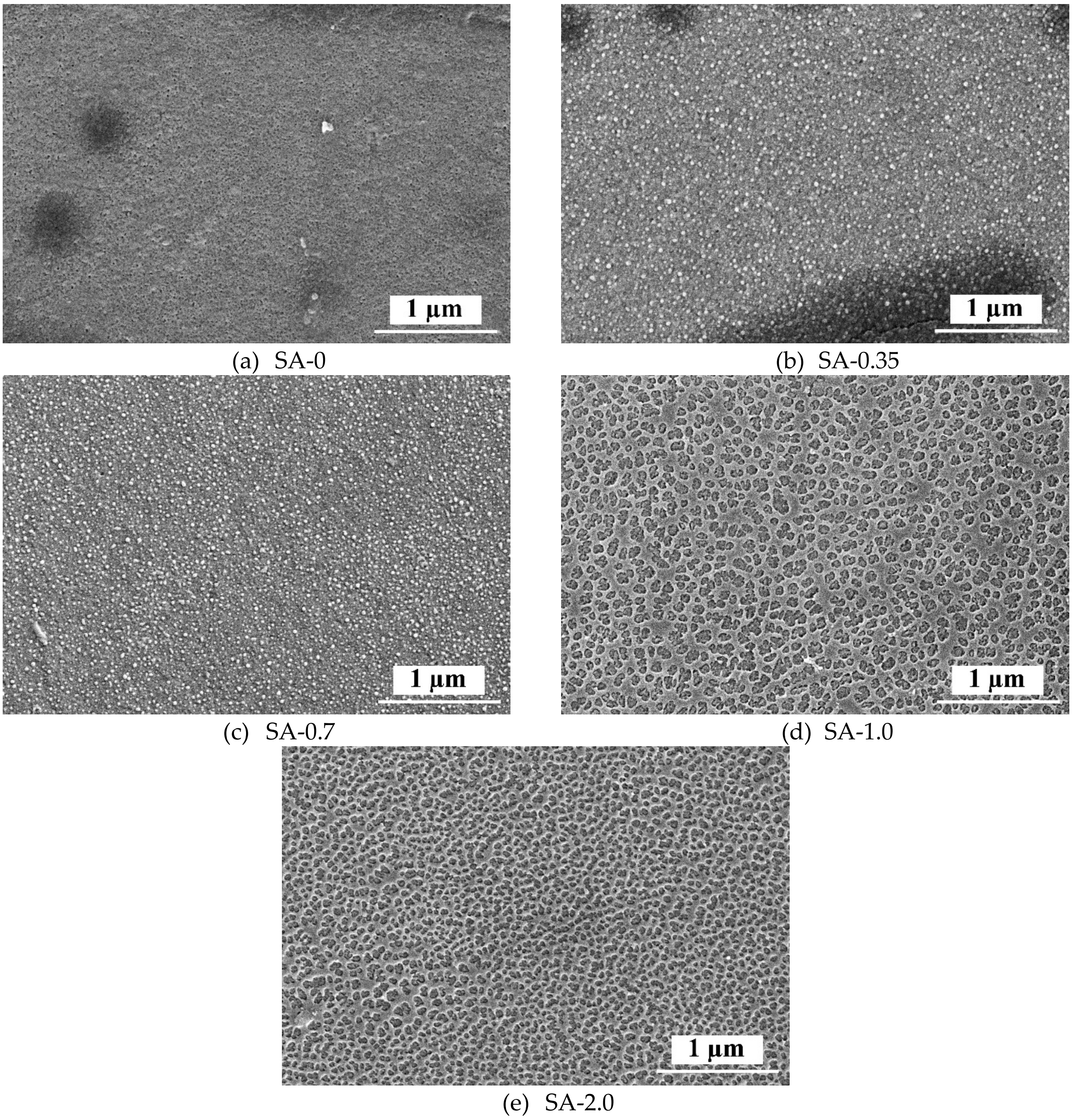

Figure S1. SEM microphotographs of the membrane selective layer surface: a – SA-0; b – SA-0.35; c – SA-0.7; d – SA-1.0; e – SA-2.0. Membrane samples were prepared by step-wise solvent exchange.
